# Editorial: Frontiers in skeletal muscle wasting, regeneration and stem cells

**DOI:** 10.3389/fphys.2015.00141

**Published:** 2015-05-13

**Authors:** Carlos H. J. Pinheiro, Lucas Guimarães-Ferreira

**Affiliations:** ^1^Department of Physiology and Biophysics, University of Sao PauloSao Paulo, Brazil; ^2^Center of Physical Education and Sports, Federal University of Espirito SantoVitoria, Brazil

**Keywords:** skeletal muscle, stem cells, satellite cells, muscle regeneration, muscle wasting

“Frontiers in Skeletal Muscle Wasting, Regeneration and Stem Cells” is a Frontiers Research Topic aimed to highlight the available knowledge regarding skeletal muscle and stem cell biology in the context of both physiological and pathological conditions. In the last decades we have many advances in the understating of muscle biology and pathophysiology of myopathies. Herein we presented articles focused in skeletal muscle biology and both pathophysiology and treatment of skeletal muscle disorders.

Animal models are frequently used in the study of muscle atrophy, focusing on its molecular mechanisms or on the search for strategies of muscle atrophy attenuation. On this regard, Baldwin et al. (2013) presented a review on the effect of unloading on the muscle phenotype mostly based on the extensive work carried out by the authors over the last 25 years using different experimental models as microgravity and hindlimb suspension, among others. Brooks and Myburgh (2014) also discuss the skeletal muscle atrophy, focusing on the interplay between myonuclei, satellite cells and signaling pathways, highlighting the multi-dimensional feature of skeletal muscle wasting. Still regarding skeletal muscle atrophy, Koopman et al. (2014) discuss recent findings linking changes in metabolism to changes in muscle stem cell function and skeletal muscle mass, discussing the “metabolic reprogramming” concept and Manring et al. (2014) address the roles of modulatory genes of the skeletal muscle excitation-contraction coupling process on muscle wasting, bringing new possibilities for the treatment of muscle diseases.

The misposition of myonuclei is a common feature of myopathies. In these conditions, nuclei are localized within the center of the muscle fiber. For decades, the centralized myonuclei was used as an evaluative parameter of regeneration in skeletal muscle. In this special issue, Dr Folker and Dr Baylies presented an interesting review regarding a possible role of myonuclei mispositioning in pathophysiology of muscle disorders (Folker and Baylies, 2013).

Regarding the treatment of muscle disorders and cachexia, Berardi et al. (2014) discussed the effect of many interventions including stem cell and gene therapies, myostatin inhibition, tumor necrosis factor alpha (TNF-alpha) and interleukin-6 (IL-6) pharmacologic antagonism and microRNAs (miRNAs). Muscle gene therapy is a very actual issue for discussion in scientific community. Where we are on this way and where we should go? Beyond the correction of gene defects in muscle cells, the therapy should consider improve capillarity and reduce fibrosis to improve muscle environment and the stem cells engraftment. On this way, Dr Bertoni presented a review pointing out that gene therapy needs to target also muscle progenitors cells (and not only mature muscle fibers) to restore the loss of myofibers as the result of the diseases progression.

Another new and exciting area explored in this issue is the role of endoplasmic reticulum (ER) stress in human skeletal muscle and its contribution to sarcopenia. Deldicque (2013) discuss this issue proposing that aging-related ER stress can impact muscle mass through cell death and creating a state of anabolic resistance by inhibiting the mTOR pathway. The author still hypothesized that exercise could reduce ER stress and can account to the beneficial effects of exercise in the elderly. This is a new exciting area and further investigations will clarify the association between ER stress and aging, and the effects of exercise.

It's well known that satellite cells are involved in skeletal muscle regeneration, which it's essential for tissue remodeling and for the cellular adaptations in response to physical exercise, specially after eccentric contractions. However, Boppart et al. (2013) present a mini-review highlighting the regulatory role for muscle-resident non-satellite stem cells in the process of muscle repair following exercise. This is an exciting new area and will stimulate further investigations to elucidate the exact role and the mechanisms of non-satellite cell-mediated muscle repair post-exercise. Regarding the satellite cells, Fukada et al. (2013) introduce the methodology of direct isolation of these cells, present a discussion about the molecular regulation mechanisms and discuss the relationship between satellite cells function and the progression of muscular disorders, as well as the potential of the satellite cells to treatment of muscle disorders. In turn, Uezumi et al. (2014) discuss the role of non-myogenic mesenchymal progenitors in skeletal muscle pathogenesis and regeneration and Meregalli et al. (2014) discuss how the advances in the isolation of new stem cells subpopulations and the creation of artificial stem cell niches can offer promises for therapeutic approaches in the treatment of muscle diseases and muscular wasting conditions.

In the present research topic, some modulators of skeletal muscle form and function are also presented and discussed. In a comprehensive perspective article, Senf (2013) reviews the experimental evidences for the biological functions of 70 kDa heat shock protein (HSP70) in skeletal muscle, with regards to its role on the muscle damage protection, muscle regeneration and recovery and muscle mass maintenance and integrity. Importantly, unanswered questions are highlighted and more information about the relation between the HSP70 protein and skeletal muscle plasticity should arise from future studies. Donati et al. (2013) review the role of sphingosine 1-phosphate (S1P) in skeletal muscle biology and homeostasis, focusing in its role on regulation of activation and proliferation of muscle-resident satellite cells, as well as on mesenchymal progenitors such as mesoangioblasts, originated outside skeletal muscle. These stem cells populations are involved in skeletal muscle repair following injury and in muscular disorders. Future studies will explore more details about the regulatory mechanisms of S1P metabolism and its precise role on skeletal muscle biology and determine the therapeutic potential of S1P signaling pathway in skeletal muscle diseases.

Another important players on the skeletal muscle plasticity are the Caspase family proteins. Caspases are important in the balance between apoptosis and regeneration, acting as a player in the maintenance of skeletal muscle structure and function. Connolly et al. (2014) present a summation of the current state of the field on the non-apoptotic roles of caspases in a range of different models, discussing the findings discovered to date, focusing on skeletal muscle. Also, in a hypothesis and theory article, Avin et al. (2014) present a review of the literature and the preliminary evidence that Klotho protein may be modulated by skeletal muscle activity and can be a link between exercise and its anti-aging effect.

The importance of skeletal muscle mass and strength, as well as its metabolic function for exercise and daily living activities is well known. Also, and not less important, alterations on skeletal muscle structure and function play a key role in many pathologic conditions and chronic diseases. On this regards, this research topic also presents articles discussing the relation between skeletal muscle and diseases such as chronic obstructive pulmonary disease (Mathur et al., 2014), diabetes (D'Souza et al., 2013) motor neuron diseases (Boyer et al., 2013), Duchenne muscular dystrophy (Bertoni, 2014) and obesity (Akhmedov and Berdeaux, 2013).

In addition to all these great contributions, we present herein original papers addressing the association of skeletal muscle hypertrophy with increases in proteasome activity independent of the ubiquitin-ligases MuRF1 and MAFbx expression (Baehr et al., 2014); the effects of docosahexaenoyl ethanolaminde on glucose uptake in proliferating and differentiating C2C12 myoblasts altering the endocannabinoid system expression (Kim et al., 2014); and the importance of the multifunctional cell surface peptidase CD13 on mesenchymal stem cell-mediated tissue repair (Rahman et al., 2014).

This research topic will provide readers with new insights and viewpoints and will stimulate new investigations and further advances in this research field. In our opinion our main objective was achieved.

## Conflict of interest statement

The authors declare that the research was conducted in the absence of any commercial or financial relationships that could be construed as a potential conflict of interest.
